# Structure-Based Development of Small Molecule PFKFB3 Inhibitors: A Framework for Potential Cancer Therapeutic Agents Targeting the Warburg Effect

**DOI:** 10.1371/journal.pone.0024179

**Published:** 2011-09-21

**Authors:** Minsuh Seo, Jeong-Do Kim, David Neau, Inder Sehgal, Yong-Hwan Lee

**Affiliations:** 1 Department of Biological Sciences, Louisiana State University, Baton Rouge, Louisiana, United States of America; 2 Northeastern Collaborative Access Team, Cornell University, Argonne, Illinois, United States of America; 3 Department of Comparative Biomedical Science, School of Veterinary Medicine, Louisiana State University, Baton Rouge, Louisiana, United States of America; University of Delhi, India

## Abstract

Cancer cells adopt glycolysis as the major source of metabolic energy production for fast cell growth. The HIF-1-induced PFKFB3 plays a key role in this adaptation by elevating the concentration of Fru-2,6-BP, the most potent glycolysis stimulator. As this metabolic conversion has been suggested to be a hallmark of cancer, PFKFB3 has emerged as a novel target for cancer chemotherapy. Here, we report that a small molecular inhibitor, N4A, was identified as an initial lead compound for PFKFB3 inhibitor with therapeutic potential. In an attempt to improve its potency, we determined the crystal structure of the PFKFB3•N4A complex to 2.4 Å resolution and, exploiting the resulting molecular information, attained the more potent YN1. When tested on cultured cancer cells, both N4A and YN1 inhibited PFKFB3, suppressing the Fru-2,6-BP level, which in turn suppressed glycolysis and, ultimately, led to cell death. This study validates PFKFB3 as a target for new cancer therapies and provides a framework for future development efforts.

## Introduction

Unlike normal cells, cancer cells have been noted to shift their energy metabolism toward glycolysis [1]. This phenomenon, originally termed the Warburg effect and this transition allows cancer cells to satisfy increased biosynthetic requirements for biomass and energy [2], [3]. Studies have consistently shown an abnormally high glycolytic rate in a wide spectrum of human cancers but the causative mechanisms responsible for this metabolic adaptation remain poorly understood [4], [5]. Among the possible mechanisms, mitochondrial respiratory defects and hypoxia in the tumor microenvironment are attributed as two major factors for the Warburg effect [6], [7], [8]. Despite the complexity and obscurity of underlying mechanisms responsible for the Warburg effect, the metabolic consequences are a consistent transformation toward glycolysis as the major source of ATP production [4], [9]. This metabolic abnormality of cancer cells provides abiochemical basis to preferentially suppress progression of malignant cells by selective inhibition of glycolysis [10], [11], [12].

In the glycolysis pathway, phosphofructokinase-1(PFK-1) catalyzes the major rate-limiting step that converts fructose-6-phosphate (Fru-6-P) to fructose-1, 6-bisphosphate (Fru-1, 6-BP) and is allosterically regulated by fructose-2,6-bisphosphate (Fru-2,6-BP) [13], [14]. Under abundant energy supply, high levels of ATP strongly inhibit PFK-1 activity; however, Fru-2,6-BP can override this inhibitory effect and enhance glucose uptake and glycolytic flux [15]. Not surprisingly, Fru-2,6-BP synthesis is up-regulated in many cancer cell lines, suggesting that selective depletion of intracellular Fru-2,6-BP in cancer cells may potentially be used to impede glycolytic flux and suppress malignant cell survival and progression [16], [17], [18].

A family of bifunctional enzymes, 6-phosphofructo-2-kinase/fructose-2,6-bisphosphatases (PFKFB1–4), are responsible for the intracellular levels of Fru-2,6-BP [18], [19], [20]. Among these isozymes, PFKFB3 is dominantly over-expressed in thyroid, breast, colon, prostatic, and ovarian tumor cell lines [18], [21], [22]. Recent studies have shown that induction of PFKFB3 expression by HIF-1 under hypoxic condition is followed by increased invasive potential and resistance to chemotherapies [21], [23]. Taken together, these studies suggest PFKFB3 is a potential target for a new class of anti-neoplastic agents that prevent onset of the cancer-specific glycolysis by inhibiting the Fru-2,6-BP surge and, eventually, induce death of cancer cells. Accordingly, inhibition of PFKFB3 as a therapeutic strategy for cancer has been suggested [22].

Despite the potential merits, exploitation of PFKFB3 for cancer therapy has remained poor. Clem et al (2008) reported a pyridinyl-containing compound as a possible PFKFB3 inhibitor, based on the receptor structure predicted from that of PFKFB4 [24]. Although promising, inhibitors based on structures other than the true PFKFB3 enzyme may lack specificity and limit strategic improvement of inhibitor potency. We were able to overcome such an inborn defect by engaging in the structural studies of PFKFB3 and its complexes with ligands. In this report, we have identified N4A as a novel competitive inhibitor and tested its inhibitory effect on PFKFB3 activity. To understand the molecular mechanism of inhibitor-recognition by PFKFB3, we determined the structure of the PFKFB3 in complex with N4A.Guided by the structural basis for inhibitor binding; we were then able to optimize N4A, using similarity search and computational evaluation, resulting in a follow-up lead compound with a 5-fold improvement in potency.

In addition to the molecular mechanism of PFKFB3 inhibition and inhibitor improvement, we also investigated the inhibition of Fru-2,6-BP production and glycolysis in HeLa cells by the PFKFB3 inhibitor treatment. The novel PFKFB3 inhibitors, N4A and YN1 reduced the Fru-2,6-BP levels and glycolytic flux, resulting in growth inhibition of tumor cells and massive cell death. These results provide not only evidence that validates targeting of PFKFB3 but also the first direct structural insight into the protein inhibitor interactions, establishing a foundation for structure-assisted optimization and development of novel PFKFB3 inhibitors as chemotherapeutic agents for cancer.

## Results

### Overall strategy for inhibitor screening and improvement

**Figure 1:**
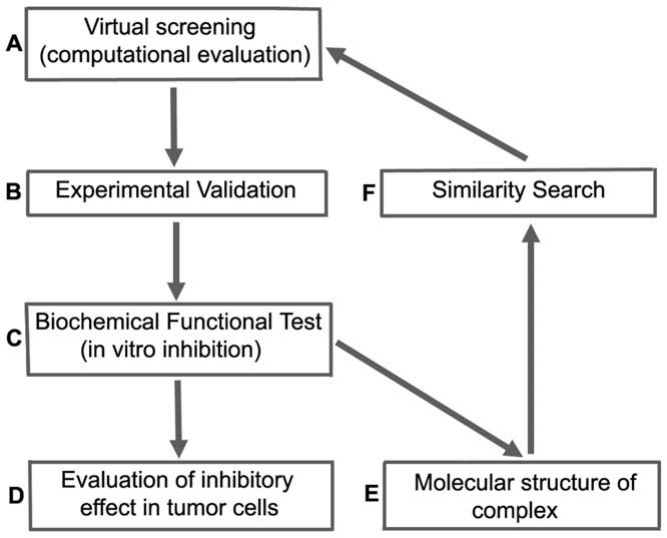
Flowchart showing the overall strategy used for development of the PFKFB3 inhibitors.

A schematic flow diagram describing our strategy adopted for discovery and improvement of the PFKFB3 inhibitors is shown in Figure 1. Candidates for a lead compound were selected from computational screening using the crystal structure of PFKFB3 which we have previously determined to 2.1 Å resolution [25] was used as molecular sieve of screening(**a**). The resulting hit compounds from this molecular sieve were evaluated by enzymatic inhibition assay and compounds with the highest inhibition activity were selected as lead molecules after consideration of drug-likeliness (**b**). Next, detailed kinetic properties were characterized (**c**) and the biological effects on human adenocarcinoma cells were investigated by measuring glycolytic flux, growth inhibition, and cell death (**d**). To understand the molecular basis of the inhibition of PFKFB3, X-ray crystallographic structure analysis of the PFKFB3•inhibitor complex was carried out (**e**). Based on the molecular information gained from Step (**e**), we performed a search for novel derivative compounds with improved potency, using the lead compound as a template (**f**). The resulting compounds from this similarity search were evaluated through computational docking using FlexX [26] (**a**) and the best optimized compound was passed through this selection process again. Through iterative cycles of these processes, we were able to obtain a compound with inhibitory activity orders of magnitude above the initial lead and which exhibits potent PFKFB3 inhibition in vitro.

### Inhibitor Screening and Binding Properties

**Figure 2:**
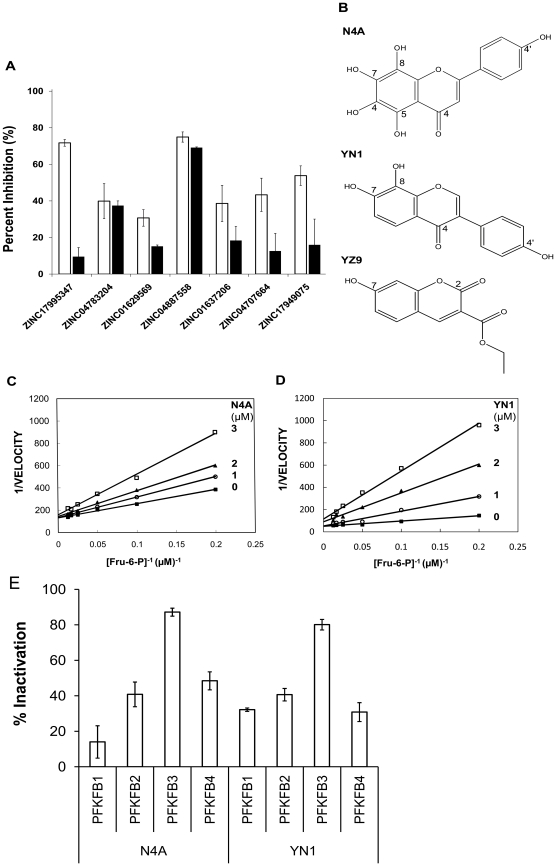
Experimental evaluation of the hit compounds. (**A**) Inhibition potencies of the candidate compounds. The magnitudes of inhibition by compounds at 10 µM each are measured through the enzyme assay and presented as percentiles against the control (□). A same experiment was also performed in the presence of 0.1% Tween-20 (▪), to eliminate false positives caused by nonspecific hydrophobic interactions. (**B**) Structures of the PFKFB3 inhibitors. (**C**) Lineweaver-Burk plots showing the competitive inhibition by N4A against Fru-6-P. The inhibitor concentrations used were: 0 µM (▪), 1 µM (○), 2 µM (▴), and 3 µM (**□**) of N4A. They are also labeled next to individual plots. (**D**) Lineweaver-Burk plots showing the competitive inhibition by YN1 against Fru-6-P. The inhibitor concentrations used were: 0 µM (▪), 1 µM (○), 2 µM (▴), and 3 µM (**□**) of N4A. (**e**) Selectivity of N4A and YN1 on PFKFB isoforms. Results are expressed as percent inhibition at twice the IC_50_ concentration against PFKFB3 (N4A = 6 µM, YN1 = 1.3 µM).

During our previous study, several compounds capable of binding to the Fru-6-P pocket of PFKFB3 were identified from virtual screening. To confirm the inhibitory activities and to eliminate false positives from these drug candidates, PFKFB3 inhibition was tested at 10 µM each of the compounds (Figure 2A). To prevent non-specific inhibition caused by random hydrophobic interactions between inhibitor and protein, a same test was performed in the presence of 0.1% Tween-20. Among the tested compounds, ZINC04887558 (N4A, 5, 6, 7, 8-tetrahydroxy-2-(4-hydroxyphenyl) chromen-4-one) inhibited enzyme activity greater than 65% under substrate-saturating conditions and this inhibition was not affected by the presence of Tween-20. We selected N4A as an initial ‘lead’ (Figure 2B). The subsequent kinetic study revealed that N4A inhibits PFKFB3 with an IC_50_ value of2.97±0.16 µM (Table 1). A steady state inhibition study showed that N4A inhibits PFKFB3 as a competitive inhibitor against Fru-6-Pwith a K_i_ of 1.29±0.26 µM, as expected from virtual screening and as demonstrated in a Lineweaver–Burk plot (Figure 2C).

**Table 1:** The kinetic and biological properties of the PFKFB3 inhibitors.

Inhibitor	IC_50_ [µM]	K_i_ [µM]	Inhibition	GI_50_[µM]
N4A	2.97±0.16	1.29±0.26	Competitive to Fru-6-P	14.2±1.5
YN1	0.67±0.08	0.24±0.03	Competitive to Fru-6-P	8.2±0.8
YZ9	0.18	0.094	Competitive to Fru-6-P	2.7±0.2

Improving the inhibition efficacy of the lead compound, N4A, by structure-guided optimization is an important goal of this study. As the details will be discussed in the following sections, only a brief end result is introduced here for early comparisons. Two additional N4A inhibitors, YN1 (7, 8-dihydroxy-3-(4-hydroxyphenyl) chromen-4-one) and YZ9 (ethyl 7-hydroxy-2-oxochromene-3-carboxylate) (Figure 2B )have been obtained using structure-guided optimization. As summarized in Table 1, YN1 exhibitsIC_50_ = 0.67 µM and K_i_ = 0.24±0.03 µM, showing a 5-fold increase in inhibition. Compound YZ9 shows even greater inhibition—one order of magnitude over the starting lead, N4A. All the tested compounds are soluble in various aqueous solutions up to 50 µM ranges in the presence of <1% dimethyl sulfoxide (DMSO).

The lead compound, N4A, and a derivative, YN1, were tested for their inhibitory effects on other human PFKFB isoforms. The inhibitors had a stronger effect on PFKFB3 than on other PFKFB isoforms. At twice the IC_50_ for PFKFB3 where PFKFB3 was over 80% inhibited, N4A exhibits less than 50% inhibition and YN1 shows less than 40% inhibition on PFKFB1, PFKFB2 and PFKFB4 (Figure 2E). N4A and YN1 are comparatively selective inhibitors of PFKFB3. Improving the isoform specificity must be the main goal of next stage optimization and such efforts are being made.

### Effects of N4A and YN1 on the Fru-2,6-BP levels, glycolysis, and cell growth

**Figure 3:**
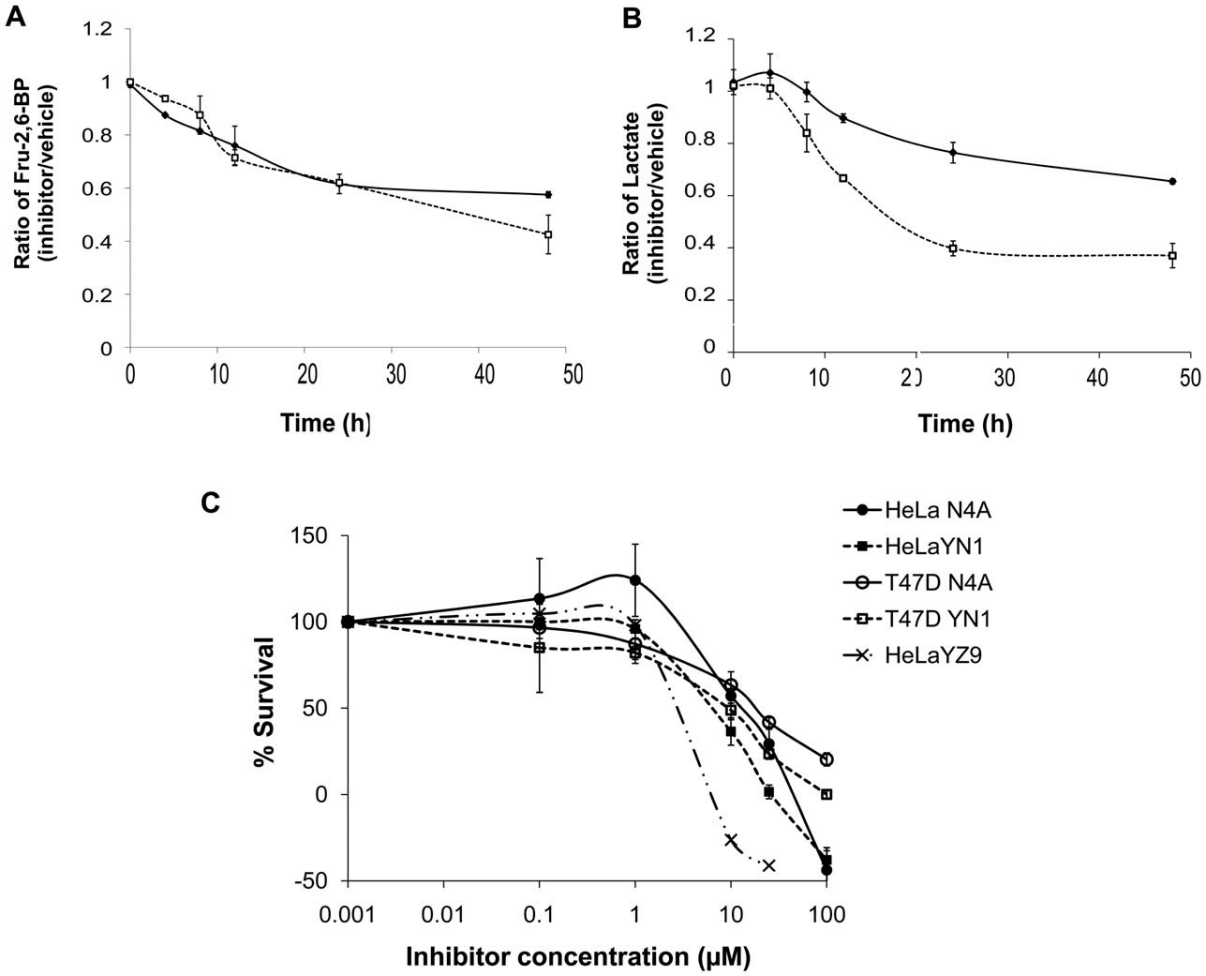
The effects of the PFKFB3 inhibitors on the Fru-2,6-BP levels, the lactate secretions, and the cell growths. The Fru-2,6-BP levels (**A**) and the levels of secreted lactate (**B**) were determined enzymatically at time points 0, 4, 8, 12, 24, or 48 hours after the inhibitor treatments of HeLa cells. The results were normalized to the sample protein concentrations and expressed as a ratio to the value of vehicle-treated. Data are means ± S.E.M. from at least three experiments. Time-dependent effects of 25 µM each of N4A (line with diamond) and YN1 (dotted line with hollow square) on the cellular Fru-2,6-BP levels (**A**) and the lactate secretions (**B**) are shown. (**C**) Growth inhibition by N4A , YN1, and YZ9 on HeLa and T47D cells. Cell numbers were assayed over 36 hours by the trypan blue counting or XTT assay. Data points are expressed as % cell growth of control containing vehicle against logarithmic scale of inhibitor concentrations. Error bars stand for intraexperimental replicates standard deviation.

We next investigated effects of applying the N4A and YN1inhibitors to live HeLa cells. Inhibition of PFKFB3 is expected to cause a decrease in the levels of Fru-2,6-BP in HeLa cells. After an 8 hour exposure to N4A and YN1, Fru-2,6-BP were reduced approximately 20%; after a 48-hour exposure, Fru-2,6-BP was reduced over 40% (Figure 3A). Down-regulation of the Fru-2,6-BP levels by N4A and YN1 was accompanied by decreased glycolysis, as expected. The decrease in the Fru-2,6-BP levels following exposure to N4A and YN1 led to a decrease in lactate production, which was reflected by a greater than 30% decrease in lactate secretions. Taken together, these data suggest N4A and YN1 inhibit PFKFB3 resulting in suppression of glycolysis (Figure 3B).

**Figure 4:**
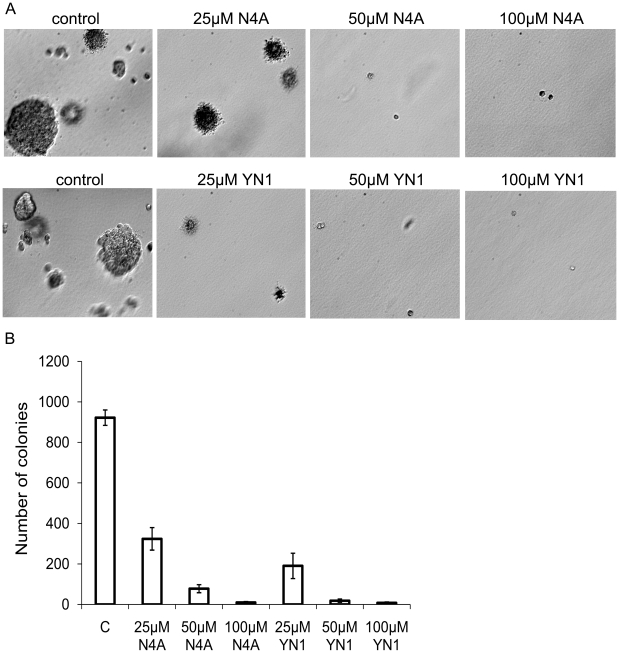
Treatment of HeLa cells with N4A and YN1 inhibits soft agar colony formation. (**A**) Anchorage-independent cell growth in soft agar. HeLa cells were grown in soft agar for 21 days in the presence of the indicated concentrations of N4A and YN1 respectively (20×). (**B**) Statistical analysis of the experiment. Columns, mean (n = 5); bars, SD.

Increased Fru-2,6-BP levels preceded by increased glycolysis often accompanies the proliferation of transformed cells, including cancer cells [3]. We examined the effect of the PFKFB3 inhibitors, N4A and YN1, on the proliferation rate of human adenocarcinoma cells. Treatments with 25 µM each of N4A and YN1 caused 70% and over 90% reductions, respectively, in cell proliferation, when compared to unexposed cells (Figure 3C). The results of cell growth inhibition assays consistently confirmed that inhibition of PFKFB3 by N4A and YN1 suppresses cellular energy metabolism and, ultimately, cell growth and that YN1 is a more potent inhibitor with a GI_50_ of 8.2±0.8 µM compared with N4A (GI_50_ = 14.2±1.5 µM) (Table 1). N4A and YN1 were also able to inhibit soft agar colony formation in HeLa cells (Figure 4). HeLa cells were plated in soft agar with different concentrations of N4A or YN1 and grown for 3 weeks to allow colony formation. Both compounds significantly inhibited colony formation at 25, 50 and 100 µM. Colony formation was inhibited by 64% and 79% in the presence of 25 µM N4A and YN1, respectively.

**Figure 5:**
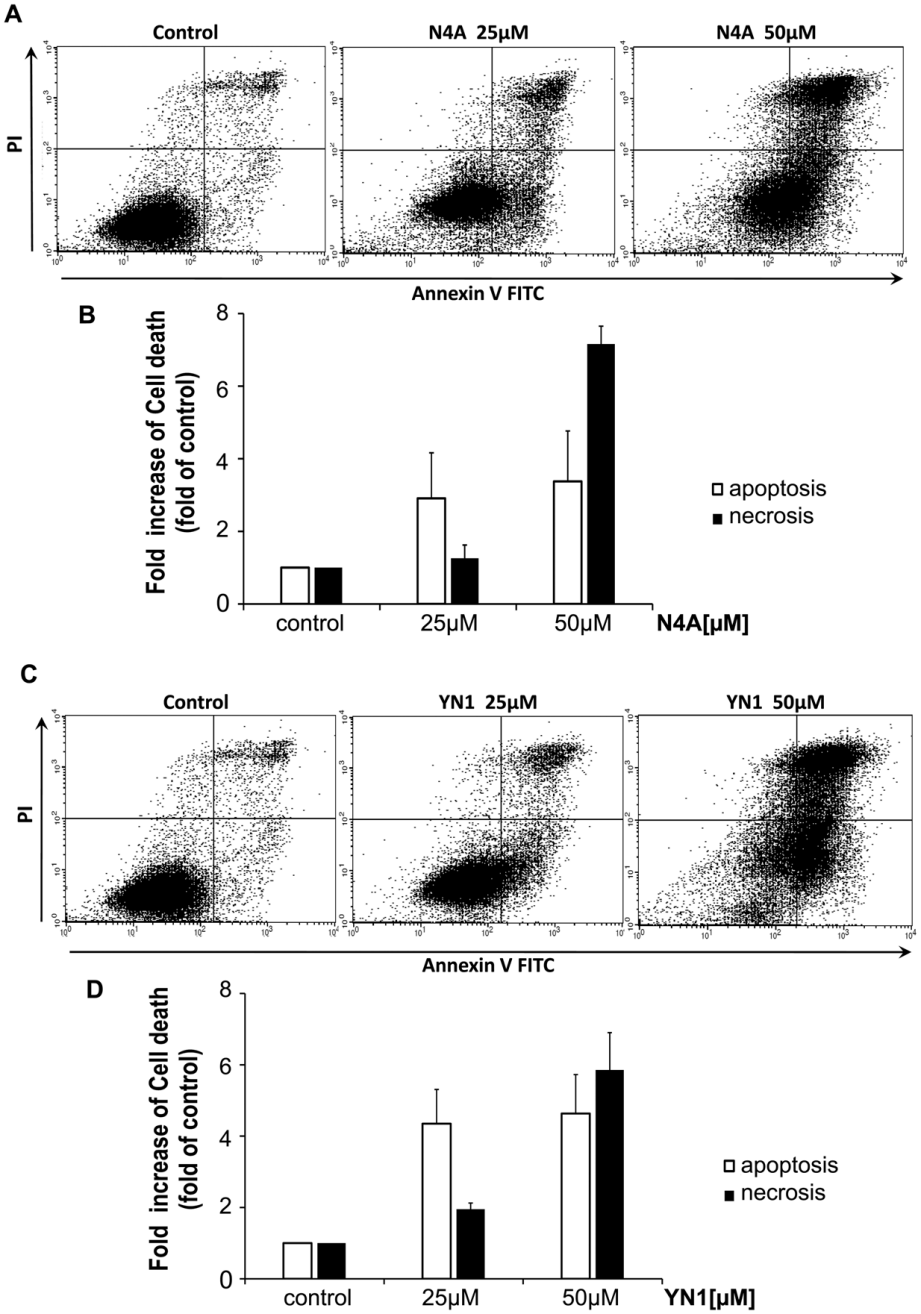
Induction of cell death in HeLa cells by the PFKFB3 inhibitors, N4A and YN1. The cells were treated with two different concentrations of inhibitors, 25 µM and 50 µM. (**A**) Induced cell death at two different concentrations of N4A was measured by flow cytometry after double staining with Annexin V and PI. (**B**) Quantitation of the flow-cytometric data (mean ± SD) showing a dose-related effect of N4A. (**C**) Cytograms of YN- induced cell death and (**D**) quantitation of the flow-cytometric data.

To further investigate the mechanism responsible for the anti-proliferative effect of the PFKFB3 inhibitors, flow-cytometric analysis of cell death was performed. The results indicate that N4A and YN1 induced both apoptotic and necrotic cell death. This mixed pattern is related to the nature of apoptosis, which, unlike necrosis, is an ATP-dependent process [27], [28]. Cell death induced by the PFKFB3 inhibitors should correlate with their ability to deplete cellular ATP and depletion of ATP favors death by necrosis as previously speculated [11], [27], [28]. Our data supports this argument: at a relatively low concentration (25 µM) of N4A or YN1, an environment in which depletion of cellular energy depletion is moderate, cells were found to be prone to apoptosis, whereas, at higher concentrations (50 µM) of inhibitors, death by necrosis was significantly increased due to insufficient cellular energy to support the apoptotic process (Figure 5).

### Structure of the PFKFB3•N4A complex

**Figure 6:**
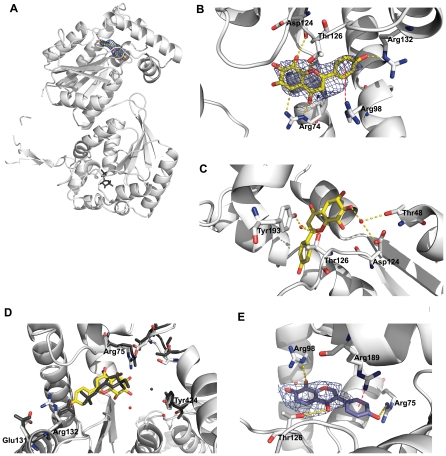
Structure of PFKFB3 in complex with inhibitors. (**A**) Ribbon diagram of the crystal structure of the PFKFB3•N4A complex. N4A bound to the PFKFB3 Fru-6-P site in the 2-Kase catalytic pocket is shown with a concomitant |F_o_|−|F_c_| omit map at a 2.5 level. The Fru-6-P bound to the 2-Pase domain is also shown in gray for comparison. (**B**)The interactions between N4A and PFKFB3 are shown. Hydrogen bonds are shown as yellow dotted lines and a Cation-π interaction is represented by a red broken line. (**C**) The water-mediated hydrogen bonds between PFKFB3 and N4A are shown in yellow dotted lines. (**D**)Inhibitor-induced local conformational changes around the N4A binding groove. Comparison of the structures of the PFKFB3•AMPPCP•Fru-6-P complex (dark gray) and the PFKFB3•N4A complex (color) was made. (**E**)YN1 bound to the same pocket with a |F_o_|−|F_c_| omit map at 2.5 level is shown. Hydrogen bonds between YN1 and PFKFB3 are shown as yellow dotted lines and Cation-π interactions as red broken lines.

We wished to use the N4A inhibitor as a lead for structure-guided optimization of further inhibitors. To facilitate this task, it was necessary to determine the molecular characteristics of N4A binding to PFKFB3 by crystallizing human PFKFB3 in the presence of N4A. We determined the structure of this complex to 2.4 Å resolution by a method of molecular replacement using the first PFKFB3 structure (PDB code: 2AXN) as a search model [29]. An |*F*_o_|−|*F*_c_| omit map enabled unambiguous placement of N4A into theFru-6-P binding pocket of the kinase domain of PFKFB3 (Figure 6A). The N4A located at the Fru-6-P pocket could be superimposed onto the Fru-6-P modeled in the structure of PFKFB3 in a ternary complex with AMPPCP and Fru-6-P (PDB code: 2DWP) [29]. This structure provides clear evidence, supporting the kinetic observations that N4A competes with Fru-6-P for the same binding pocket in PFKFB3 (Figure 6B, C, D). As shown in Figure 6B and D, N4A is anchored to the Fru-6-P pocket via hydrogen bonds with Arg74, Asp124, Thr126, and Arg132. The phenol moiety of N4A is unfavorably located in the pocket where the 6-phosphate moiety of Fru-6-P interacts through a hydrogen bond with Arg132 and results in Arg132 adopting a different conformation in the N4A complex. The chromone moiety of N4A occupies the same position as the fructose moiety. Here, two of N4A's hydroxyl groups (OAF and OAA) mimic the hydrogen-bonding pattern of the hydroxyl groups at the C3 and C4 positions of the fructose moiety. The binding competence of N4A is further strengthened by a number of water-mediated hydrogen bonds between the hydroxyl group of the N4A with Thr48, Arg98, Asp124, Thr126, and Tyr193 and the conformational changes induced upon the binding (Figure 6C). The protein ligand interactions are summarized in Table 2.

**Table 2:** Interactions between the inhibitors and PFKFB3.

Protein residue		Water	Inhibitor N4A	Type of interaction	Distance (Å)
Arg74	NE		O4	Hydrogen bond	2.9
	NH2		O5	Hydrogen bond	3.0
Asp124	O		O8	Hydrogen bond	3.5
Thr126	OG1		O1	Hydrogen bond	3.3
Arg132	NE		O4′	Hydrogen bond	2.2
Arg98			Phenol moiety	Cation-π interaction	5
Thr48	OG1	HOH38			3.3
Asp124	OD1	HOH38			3.2
		HOH38	O7	Water mediated interaction	2.3
Thr126	OG1	HOH97			3.5
Tyr193	OH	HOH97			2.2
		HOH97	O1	Water mediated interaction	2.6

Protein residue		Water	Inhibitor YN1	Type of interaction	Distance (Å)
Arg75	NH1		O4′	Hydrogen bond	3.5
Arg98	NE		O8	Hydrogen bond	3.2
Thr126	OG1		O4	Hydrogen bond	3.5
Arg98			Chromone moiety	Cation-π interaction	4
Arg189			Phenol moiety	Cation-π interaction	3.5

When compared with PFKFB3 complexed with AMPPCP and Fru-6-P (PDB code: 2DWP), the N4A-PFKFB3 complex induces no significant difference in global structure. However, the differences around the F-6-P binding pocket were evident as shown in Figure 6D. Upon N4A binding, the Arg132 side chain swings out about 3.5 Å from the position for Fru-6-P site, offering space to accommodate N4A. As a consequence, Glu131 in the same helix moves toward the F-6-P pocket by ∼2 Å. The guanidine group of Arg75 moved 1.5 Å toward the inhibitor from its original position, implying that the closer positioning of Arg75 stabilizes inhibitor binding. The phenol group on Tyr424 is tilted toward N4A due to the repositioning of water molecules near Tyr424 upon N4A binding. These local conformational changes in the F-6-P pocket affect the mobile ATP loop, especially a turn (residues 168–180), which flips into the F-6-P pocket, a displacement of over 2 Å (data not shown).

### YN1 and YZ9 from the exploration of N4A scaffold

#### YN1

To increase the inhibition potency of N4A, firstly, we performed a similarity search using N4A as a template and with a defined Tanimoto coefficient ≥0.9 [30]. The selected molecules from the entire NCI Database were evaluated via computational docking [26] and, as a result, ZINC06093399 (YN1) was predicted to have significantly improved potency as a PFKFB3 inhibitor. YN1, purchased from CHESS GmbH, was tested for its inhibition potency and kinetic properties and demonstrated a 5-fold increase in PFKFB3 inhibition compared to N4A (Table 1).

In order to determine the molecular basis for the increased inhibition potency of YN1, the crystal structure of the PFKFB3 in complex with YN1 was also determined but at the modest resolution of 3.3 Å. Because of this resolution limit, the structure refinement was performed using only rigid body and B-group refinement, taking the whole protein structure of the N4A complex as a rigid body. Similar to N4A, YN1 binds to theFru-6-P binding pocket as clearly shown in an omit |*F*_o_|−|*F*_c_| electron density maps (Figure 6E). In addition, like N4A, YN1 interacts with PFKFB3 by occupying the Fru-6-P pocket. However, the electron density for YN1 is better modeled by flipping YN1 180° in respect to its short axis, compared to the orientation of N4A, resulting in the phenol moiety of YN1 being positioned towards the site occupied by the fructose moiety of Fru-6-P rather than the site of the phosphate of Fru-6-P. This orientation may be a consequence of the substitution of benzenediol for benzenetetrol in the chromone moiety of the two inhibitors. The additional hydroxyl groups on the chromone moiety of N4A perhaps cannot be accommodated at the binding site for the 6-phosphate moiety of Fru-6-P. However, YN1 with a loss of two hydroxyl groups in the same chromone moiety is able to bind to the site in a direction opposite that of N4A. Then, the chromone moiety of YN1 is inserted between Val70 and Phe87 gaining hydrophobic interactions and a Cation-π interaction with Arg98, while its phenol moiety gains a Cation-π interaction with Arg189 and a hydrogen bond with Arg75 (Table 2). Water mediated interactions, similar to those observed in the N4A complex, very likely contribute to the YN1 interaction, although the resolution limit did not allow for the modeling of water in the YN1 complex.

Certainly, our observations regarding YN1 require supporting evidence from high resolution X-ray data. Nevertheless, because only the position of YN1 was refined inside the N4A binding pocket as a rigid body, this new binding mode observed for YN1 is significant. Comparison of the binding modes of N4A and YN1 suggested that the Fru-6-P pocket of PFKFB3, with a number of Arg residues present, is surprisingly generous to the binding of compounds with hydrophobic rings. It is likely that the pocket takes advantage of Cation-π interactions as seen in other structures [31] and water-mediated hydrogen bonds. The higher potency of YN1 compared to N4A and the apparent difference in binding modes between the two compounds, taken together, suggest that compounds containing a chromone moiety with fewer hydroxyl groups will be more potent than either N4A or YN1.

#### YZ9

Using a strategy similar to that used to find YN1, we identified YZ9. Using the same biological test routine, we determined that YZ9 inhibited PFKFB3 with an IC_50_ of 0.183 µM, and acted as a competitive inhibitor against Fru-6-P with a K_i_ of 0.094 µM (Table 1). YZ9 also inhibited the cell growth with a GI_50_ of 2.7 µM. Further characterization of YZ9 is on-going.

## Discussion

Suppression of anaerobic glycolysis has been suggested as a promising strategy for the development of chemotherapeutic agents for cancer, because tumor cells exhibit an abnormally high glycolytic rate even in the presence of oxygen [10], [11], [12]. The rate of glycolysis is regulated by the cellular concentration of Fru-2,6-BP, a potent stimulator of glycolysis [13], [14], [15]. Recent studies invariably show that Fru-2,6-BP production is increased in transformed cell lines due to the expression of PFKFB3, the hypoxia-inducible isoform of the 6-phosphofructo-2-kinase/fructose-2,6-bisphosphatases, that catalyzes the synthesis of Fru-2,6-BP at least ten times greater rate than other isoforms [17], [18], [20], [21], [22]. Accordingly, it has been hypothesized that inhibition of PFKFB3 would deprive tumor cells of energy sources necessary for proliferation and growth [12], [22].

In this report, we were able to introduce a potent competitive inhibitor of PFKFB3, N4A with an IC_50_ of 2.97 µM. The inhibitor also shows a stronger effect on PFKFB3 than on other PFKFB isoforms. Despite high homology between PFKFB isoforms, the inhibitor has much higher specificity for PFKFB3 than we thought. As a result of proper targeting, the PFKFB3 inhibitor reduced the Fru-2,6-BP level and glycolytic rate in cell, ultimately, leading to tumor growth inhibition and massive cell death. The cell death induced by the inhibitor involves both apoptosis and necrosis. Our observation is coincident with previous reports that depletion of cellular energy tends to cause necrotic cell death, because apoptosis is an energy requiring process [11], [27], [28]. Supporting this idea, we found apoptotic cell death was primarily observed at a relatively low inhibitor concentration (25 µM), which would produce only moderate energy depletion. On the other hand, higher concentration of inhibitors (50 µM) significantly increased both necrosis and apoptosis, leading to an insufficient energy state where the apoptotic process is not favored.

Although N4A and YN1 show comparatively selective PFKFB3 inhibition between the PFKFB isoforms, the anti-proliferative effect of the inhibitors on cancer cells cannot be solely ascribed to the inhibition of PFKFB3 kinase activity. However, the development of N4A and YN1 is a first step toward the goal to obtain specific PFKFB3 inhibitors possessing high selectivity and low general toxicity.

Determination of protein structures in complex with an inhibitor is necessary to obtain mechanisms for inhibitor recognition at the molecular level and to provide an opportunity to identify alternative molecules with higher potency [32], [33]. Understanding the molecular basis for interactions between a potential drug molecule and its target protein is a critical step in successful drug development [33]. We were able to determine the first molecular structure of PFKFB3 in complex with an inhibitor, and then use this structural data to improve potency of the inhibitor. The crystal structures of PFKFB3 in complex with N4A revealed information on inhibitor binding at the molecular level. The crystal structure with YN1 provided an insight into alternative bindings of similar compounds, despite the moderate resolution. The two together provided the blue print of new compounds and rational guidelines for design of novel PFKFB3 inhibitors. Although YN1 is a derivative of the lead compound N4A and the binding modes of the two inhibitors are in approximately the same plane, it appears, even at the modest resolution of the YN1 complex, that the interactions of these two compounds with PFKFB3 are quite different. The difference of orientations within the same Fru-6-P plane is likely the consequence of YN1 having a less bulky chromone moiety (two fewer hydroxyl groups compared to N4A). The new binding mode observed for YN1 results in new hydrophobic interactions and the addition of Cation-π interactions, which together compensate for the loss of hydroxyl groups, which participated in hydrogen bond in the N4A complex, and appear to support the higher inhibitory activity of YN1. It is noteworthy that our inhibitors with no negatively charged group efficiently target the Fru-6-P pocket, which is populated with positively charged residues. The accompanied energy penalty for such bindings is likely to be paid by increases in the Cation-π interactions. This speculation was tested with the third compound YZ9, which showed the inhibition potency increased by an order of magnitude, although detailed characterizations including a structural analysis have yet to be performed.

Our data suggested the approach taken in this report will enable the rational design of PFKFB3 inhibitors, which will have a higher specificity by targeting the Fru-6-P site instead of targeting the ATP site since the ATP site fold is shared by thousands of other kinases [34], [35]. This report provides a framework for the rational development of the PFKFB3 inhibitors as new cancer therapeutics.

## Materials and Methods

### 2-kase assay and kinetic analysis

To determine steady-state initial reaction rates, the 2-Kase reactions were performed first and the Fru-2,6-BP produced was measured by a conventional enzyme-coupled kinetics assay as described previously [29], [36]. Initial rates of decrease in absorbance (Abs) at 340 nm were corrected with the rate of the control reaction in which no Fru-2,6-BP was present. Negative controls were carried out in the absence of enzyme and positive controls indicate the reaction in the presence of enzyme without inhibitor compounds. The percentage of Inhibitory activity was calculated according to the formula: % inhibition = 100×[1−(Abs_negativecontrol_−Abs_compound_)/(Abs_negativecontrol_−Abs_positivecontrol_)]. IC_50_ values were determined in quadruplicate. For the kinetic studies, concentrations of one of substrate and inhibitor were varied and decrease in absorbance at 340 nm was measured. The inhibition patterns were analyzed using the program written by Cleland, in which K_i_ is the dissociation constants for the inhibitor from enzyme-inhibitor complex [37]. For Selectivity studies, the 2-Kase assays were performed first and the F-2,6-P2 produced was measured by an enzyme coupled kinetics assay as described above. The His6-tagged PFKFB isoforms, PFKFB1, PFKFB2, and PFKFB4 were expressed in Escherichia coli C41(DE3) and purified using Ni-NTA affinity columns.

### Similarity Search

Similarity searches, using N4A or YN1 as a template, were performed using the NCI Enhanced Web Browser (http://129.43.27.140/ncidb2/) with a defined Tanimoto coefficient ≥0.9 [30]. The entire Open NCI Database was used using Tanimoto index and the selected compounds were consequently evaluated through docking. Docking was performed on the structure of the PFKFB3•ADP•F6P complex (PDB code: 2AXN), using FlexX, after stripping the ligands [26], [29]. The docking calculations were performed with an active-site encompassing a sphere of 15 Å around the reference structure position in the PDB file. The high rank compounds were purchased from CHESS GmbH (Germany) or from other suppliers.

### Preparation and crystallization of PFKFB3

Preparation of the protein sample and its crystallization was performed as described [25]. The 6×His-tagged human PFKFB3 was expressed in Escherichia coli BL21(DE3) pLysS and purified using Ni-NTA affinity columns and, subsequently, Mono Q anion-exchange chromatography. The resulting pure protein was kept, after concentrating to 8 mg ml^−1^ protein, in 20 mM Tris•HCl (pH 8.0), 10 mM NaP_i_, 0.05 mM EDTA, 5 mM β-mercaptoethanol, 5% glycerol, 0.2 mM Fru-6-P. Crystals were prepared by the sitting-drop, vapor-diffusion method with a 1∶1 (v/v) mixture of the protein sample with a reservoir solution of 50 mM HEPES pH 7, 7–20% DMSO, 0.2–1.5 mM of inhibitors, and 7% (w/v) polyethylene glycol 4000. Crystals with a size of 0.2 mm×0.1 mm×0.2 mm grew in two to three weeks.

### Cell culture

HeLa and T47D cell lines were cultured in a 10% CO_2_ humidified atmosphere at 37°C as exponentially growing monolayers in Dulbecco's modified Eagle's medium (DMEM) with glutamax (Invitrogen), supplemented with 10% fetal calf serum (Invitrogen) and penicillin/streptomycin (100 U/ml and 100 µg/ml). The human cervical carcinoma cell line, HeLa, was kindly provided by Dr. J. Kim (Louisiana State University) and the human breast carcinoma cell line, T47D, was obtained from the American Type Culture Collection (Manassas, VA).

### Metabolite determination

HeLa cells were plated at a density of 2.5×10^5^ in a 6-well plate in DMEM containing 10% FCS. The media were replaced with fresh DMEM containing either vehicle (dimethyl sulfoxide) or 25 µM of inhibitor the following day. After 0, 4, 8, 12, 24, or 48 hours of incubation with each inhibitor, media samples were collected for measuring the lactate secretion levels using a lactate oxidase-based assay kit (Sigma-Aldrich) and the lactate concentration was normalized to the total cellular protein concentration. The Fru-2,6-BP level was determined with collected cells at times of 0, 4,8,12,24, or 48 hours after the treatment, using the method described previously [36].

### Cell Proliferation/Survival Assays

Cell growth inhibition was determined by an XTT-based in-vitro toxicology assay (Sigma-Aldrich) or trypan blue staining. Cells were plated at a density of 3.5×10^4^ per well in a 24-well plate in DMEM containing 10% FCS. These cells were allowed to attach for 24 hours, and the media were replaced with fresh media containing either vehicle (dimethyl sulfoxide) or appropriate concentrations of test compounds. After 36 hours of incubation with either vehicle or compounds, cells were trypsinized and cell viability was determined by the trypan blue exclusion assay using a hemacytometer. For the XTT assay, cells were seeded into 96-well cell culture plates at a density of 0.6×10^4^ per well. After the appropriate treatment described above, cells were incubated with 0.1 mg/ml of 2,3-bis(2-methoxy-4-nitro-5-sulfophenyl)-5-[(phenylamino)carbonyl]-2H-tetrazolium hydroxide (XTT) for 4 hours and then cell number was determined by the absorbance at 470 nm, which is proportional to the number of cells that remained attached to the plate. GI_50_s were calculated as the inhibitor concentration needed for 50% of vehicle-treated cell growth. The appearance of apoptotic or necrotic cells was determined by flow cytometric analysis of cells double stained with Annexin V-FITC and propidium iodide (PI) [38]. Apoptotic and necrotic cells were distinguished on the basis of double-labeling for Annexin V-FITC (Sigma-Aldrich) and PI, a membrane-impermeable DNA stain. Floating and freshly trypsinized cells were pooled, washed twice in binding buffer, and processed following manufacturer's instructions. The fluorescence of samples were analyzed by flow cytometry (FACSCalibur®, Becton Dickinson Immunocytometry, San Jose, CA) using the CellQuest (BD Bioscience, Immunocytometry Systems, San Jose, CA, USA) software.

For HeLa cell colony formation was determined by soft agar assay. Cells (2×103) were mixed in DMEM medium containing 0.35% agarose and varying concentrations of N4A or YN1. Then the cell mixture was added on a layer of 0.6% concentration of bottom agar in 24 well plates and allowed to grow for three weeks at 37°Cunder 5% CO_2_. Fresh medium containing the inhibitors or vehicle (DMSO) was changed every four days. Colonies of 50 cells or more were counted after three weeks. The experiments were repeated at least five times.

### Diffraction data collection and processing

Crystals were soaked with cryoprotectant solutions for 0.5 to 2 hours before cryogenic data collections. Depending on the experimental aims, cryoprotectant solutions (a crystallization reservoir solution enriched with 30% glycerol) were enriched with 0.3 mM of inhibitor. A soaked crystal was flash-frozen at 100 K using an Oxford cryo-device and kept at the same temperature during data collections. The diffraction data were collected at The Northeastern Collaborative Access Team (NE-CAT) Beamline at the Advanced Photon Source, Argonne National Laboratory, Argonne, IL. The X-ray source wavelength was 0.9792 Å. The data recorded on a ADSC Q315 detector were integrated, merged, and scaled using XDS [39]. Statistics of the diffraction data and structure refinement are summarized in Table 1. The crystals belong to *P*6_5_22 space group with similar cell dimensions.

### Structure determination and refinement

The reduced data were formatted for the program suites of CCP4 [40] and 10% of the data were marked for free R-factor measurements in subsequent structure refinements. The search model was built from the coordinates of the PFKFB3•Fru-6-P•EDTA complex structure (PDB accession code 2AXN) [29] by stripping all the included ligand and solvent molecules to determine the N4A complex structures of PFKFB3. The initial model was determined using REFMAC within the CCP4 suit and processed through iterated cycles of manual model rebuilding and validation using the program COOT [40]. Binding of the ligands was confirmed, referring to the |Fo|−|Fc| omit maps that were generated, when R_crys_/R_free_ reached 0.23/0.29 or below. Referring to these maps, Fru-6-P, N4A or YN1 was incorporated into the corresponding complex models. As summarized in Table 3, the final model of the PFKFB3•Fru-6-P•N4A complex has Rcrys/Rfree of 0.215/0.262 using a total of 3881 scatterers, including solvent molecules, against all available 26,883 reflections in the resolution range of 61.6–2.4 Å. The structure contains a total of 443 amino acid residues of the full-length protein of 520 residues. As in the PFKFB3•ADP•EDTA complex, the C terminus (residues 446–520) is mostly disordered.

**Table 3:** Statistics of reflection data and structure refinements.

	PFKFB3·Fru-6-P·N4A	PFKFB3·Fru-6-P·YN1
Space group	*P*6_5_22	*P*6_5_22
Unit cell dimensions		
a = b (Å)	101.70	102.78
c (Å)	258.61	260.10
Resolution range (Å)	61.6-2.4	50.4-3.3
No. reflections used	26,883	20,476
Completeness (%)	100.00	88.96
Redundancy	7.2 (2.6)	9.5 (2.2)
I/σ (*I*)	10.03	6.2
R_sym_	0.055	0.069
R_crys_	0.215	0.243
R_free_	0.262	0.250
No. amino acids	443	441
No. protein atoms	3881	3637
No. hetero atoms	52	40
No. water molecules	192	-
r.m.s.d. from ideal		
Bond lengths (Å)	0.021	-
Bond angles (deg.)	2.016	-
Dihedral angles (deg.)	20.9	-
Mean *B*factor		
Protein atoms (Å^2^)	35.53	90.93
Hetero atoms (Å^2^)	48.23	106.73
Water atoms (Å^2^)	43.08	-

*R*_sym_ = ∑*_h_*(∑*_j_*|*I_h,j_*−<*I_h_*>|/∑*_Ih,j_*), where *h* = set of Miller indices, *j* = set of observations of reflection *h*, and <*I_h_*> = the mean intensity. *R*_crys_ = ∑*_h_*||*F*_o,*h*_|−|*F*_c,*h*_||/∑*_h_*|*F*_o,*h*_|. *R*_free_ was calculated using 10% of the complete data set excluded from refinement. The numbers in parentheses represent values from the highest resolution shell.

The PFKFB3•Fru-6-P•YN1 complex was built from N4A complex through only rigid body and B-group refinement because of resolution limit, resulting with R_free_/R_crys_ of 0.243/0.250 using a total of 3700 scatterers against all available 12,140 reflections in the resolution range of 89.0–3.3 Å. The structure contains a total of 448 amino acid residues of the full length protein of 520 residues. In the both structures, more than 89% of the residues are in the most favored region, 9.5% in the additional region, and the rest in the generously allowed region in the Ramachandran plots. The structure refinement statistics are summarized in Table 3.

## References

[1] Jones RG, Thompson CB (2009). Tumor suppressors and cell metabolism: a recipe for cancer growth.. Genes Dev.

[2] Warburg O (1956). On the origin of cancer cells.. Science.

[3] Vander Heiden MG, Cantley LC, Thompson CB (2009). Understanding the Warburg effect: the metabolic requirements of cell proliferation.. Science.

[4] Garber K (2006). Energy deregulation: licensing tumors to grow.. Science.

[5] Hsu PP, Sabatini DM (2008). Cancer cell metabolism: Warburg and beyond.. Cell.

[6] Polyak K, Li Y, Zhu H, Lengauer C, Willson JK (1998). Somatic mutations of the mitochondrial genome in human colorectal tumours.. Nat Genet.

[7] Gottlieb E, Tomlinson IP (2005). Mitochondrial tumour suppressors: a genetic and biochemical update.. Nat Rev Cancer.

[8] Pouyssegur J, Dayan F, Mazure NM (2006). Hypoxia signalling in cancer and approaches to enforce tumour regression.. Nature.

[9] DeBerardinis RJ, Lum JJ, Hatzivassiliou G, Thompson CB (2008). The biology of cancer: metabolic reprogramming fuels cell growth and proliferation.. Cell Metab.

[10] Pelicano H, Martin DS, Xu RH, Huang P (2006). Glycolysis inhibition for anticancer treatment.. Oncogene.

[11] Xu RH, Pelicano H, Zhou Y, Carew JS, Feng L (2005). Inhibition of glycolysis in cancer cells: a novel strategy to overcome drug resistance associated with mitochondrial respiratory defect and hypoxia.. Cancer Res.

[12] Tennant DA, Duran RV, Gottlieb E (2010). Targeting metabolic transformation for cancer therapy.. Nat Rev Cancer.

[13] Pilkis SJ, Claus TH, Kurland IJ, Lange AJ (1995). 6-Phosphofructo-2-kinase/fructose-2,6-bisphosphatase: a metabolic signaling enzyme.. Annu Rev Biochem.

[14] El-Maghrabi MR, Noto F, Wu N, Manes N (2001). 6-phosphofructo-2-kinase/fructose-2,6-bisphosphatase: suiting structure to need, in a family of tissue-specific enzymes.. Curr Opin Clin Nutr Metab Care.

[15] Van Schaftingen E, Jett MF, Hue L, Hers HG (1981). Control of liver 6-phosphofructokinase by fructose 2,6-bisphosphate and other effectors.. Proc Natl Acad Sci U S A.

[16] Nissler K, Petermann H, Wenz I, Brox D (1995). Fructose 2,6-bisphosphate metabolism in Ehrlich ascites tumour cells.. J Cancer Res Clin Oncol.

[17] Hue L, Rousseau GG (1993). Fructose 2,6-bisphosphate and the control of glycolysis by growth factors, tumor promoters and oncogenes.. Adv Enzyme Regul.

[18] Telang S, Yalcin A, Clem AL, Bucala R, Lane AN (2006). Ras transformation requires metabolic control by 6-phosphofructo-2-kinase.. Oncogene.

[19] Rider MH, Bertrand L, Vertommen D, Michels PA, Rousseau GG (2004). 6-phosphofructo-2-kinase/fructose-2,6-bisphosphatase: head-to-head with a bifunctional enzyme that controls glycolysis.. Biochem J.

[20] Minchenko O, Opentanova I, Caro J (2003). Hypoxic regulation of the 6-phosphofructo-2-kinase/fructose-2,6-bisphosphatase gene family (PFKFB-1–4) expression in vivo.. FEBS Lett.

[21] Atsumi T, Chesney J, Metz C, Leng L, Donnelly S (2002). High expression of inducible 6-phosphofructo-2-kinase/fructose-2,6-bisphosphatase (iPFK-2; PFKFB3) in human cancers.. Cancer Res.

[22] Calvo MN, Bartrons R, Castano E, Perales JC, Navarro-Sabate A (2006). PFKFB3 gene silencing decreases glycolysis, induces cell-cycle delay and inhibits anchorage-independent growth in HeLa cells.. FEBS Lett.

[23] Minchenko A, Leshchinsky I, Opentanova I, Sang N, Srinivas V (2002). Hypoxia-inducible factor-1-mediated expression of the 6-phosphofructo-2-kinase/fructose-2,6-bisphosphatase-3 (PFKFB3) gene. Its possible role in the Warburg effect.. J Biol Chem.

[24] Clem B, Telang S, Clem A, Yalcin A, Meier J (2008). Small-molecule inhibition of 6-phosphofructo-2-kinase activity suppresses glycolytic flux and tumor growth.. Mol Cancer Ther.

[25] Kim SG, Manes NP, El-Maghrabi MR, Lee YH (2006). Crystal structure of the hypoxia-inducible form of 6-phosphofructo-2-kinase/fructose-2,6-bisphosphatase (PFKFB3): a possible new target for cancer therapy.. J Biol Chem.

[26] Gohlke H, Hendlich M, Klebe G (2000). Knowledge-based scoring function to predict protein-ligand interactions.. J Mol Biol.

[27] McConkey DJ (1998). Biochemical determinants of apoptosis and necrosis.. Toxicol Lett.

[28] Richter C, Schweizer M, Cossarizza A, Franceschi C (1996). Control of apoptosis by the cellular ATP level.. FEBS Lett.

[29] Kim SG, Cavalier M, El-Maghrabi MR, Lee YH (2007). A direct substrate-substrate interaction found in the kinase domain of the bifunctional enzyme, 6-phosphofructo-2-kinase/fructose-2,6-bisphosphatase.. J Mol Biol.

[30] Perez JJ (2005). Managing molecular diversity.. Chem Soc Rev.

[31] Beene DL, Brandt GS, Zhong W, Zacharias NM, Lester HA (2002). Cation-pi interactions in ligand recognition by serotonergic (5-HT3A) and nicotinic acetylcholine receptors: the anomalous binding properties of nicotine.. Biochemistry.

[32] Kuntz ID (1992). Structure-based strategies for drug design and discovery.. Science.

[33] Blundell TL, Jhoti H, Abell C (2002). High-throughput crystallography for lead discovery in drug design.. Nat Rev Drug Discov.

[34] Davies SP, Reddy H, Caivano M, Cohen P (2000). Specificity and mechanism of action of some commonly used protein kinase inhibitors.. Biochem J.

[35] Hanks SK, Quinn AM, Hunter T (1988). The protein kinase family: conserved features and deduced phylogeny of the catalytic domains.. Science.

[36] Van Schaftingen E, Lederer B, Bartrons R, Hers HG (1982). A kinetic study of pyrophosphate: fructose-6-phosphate phosphotransferase from potato tubers. Application to a microassay of fructose 2,6-bisphosphate.. Eur J Biochem.

[37] Cleland WW (1979). Statistical analysis of enzyme kinetic data.. Methods Enzymol.

[38] van Engeland M, Nieland LJ, Ramaekers FC, Schutte B, Reutelingsperger CP (1998). Annexin V-affinity assay: a review on an apoptosis detection system based on phosphatidylserine exposure.. Cytometry.

[39] Kabsch W (2010). Xds.. Acta Crystallogr D Biol Crystallogr.

[40] Collaborative Computational Project (1994). The CCP4 suite: programs for protein crystallography.. Acta Crystallogr D Biol Crystallogr.

